# Visual augmentation of deck-landing-ability improves helicopter ship landing decisions

**DOI:** 10.1038/s41598-022-26770-2

**Published:** 2023-03-29

**Authors:** Mathieu Thomas, Julien R. Serres, Thomas Rakotomamonjy, Franck Ruffier, Antoine H. P. Morice

**Affiliations:** 1 ONERA, DTIS, Salon, Cedex Air, 13661 Salon-de-Provence, France; 2 Aix-Marseille University, CNRS, ISM, 13009 Marseille, France

**Keywords:** Psychology, Human behaviour

## Abstract

When attempting to land on a ship deck tossed by the sea, helicopter pilots must make sure that the helicopter can develop sufficient lift to be able to safely touchdown. This reminder of affordance theory led us to model and study the affordance of deck-landing-ability, which defines whether it is possible to land safely on a ship deck depending on the helicopter's available lift and the ship’s deck heave movements. Two groups of participants with no piloting experience using a laptop helicopter simulator attempted to land either a low-lifter or a heavy-lifter helicopter on a virtual ship deck by either triggering a pre-programmed lift serving as the descent law if it was deemed possible to land, or aborting the deck-landing maneuver. The deck-landing-ability was manipulated by varying the helicopter's initial altitude and the ship's heave phase between trials. We designed a visual augmentation making visible the deck-landing-ability, and thus enabling participants to maximize the safety of their deck-landing attempts and reduce the number of unsafe deck-landing. The visual augmentation presented here was perceived by participants as a means of facilitating this decision-making process. The benefits were found to have originated from the clear-cut distinction it helped them to make between safe and unsafe deck-landing windows and the display of the optimal time for initiating the landing.

## Introduction

Ship deck landing is a risky, demanding helicopter maneuver which involves reaching the deck despite the heaving and rolling of the ship, airwake perturbances, and often stressful contexts. The key factors underlying pilots’ safety and performances have therefore been extensively studied^1,2,3,4,5^. Several research teams (such as Tu-Deft^6^, University of Liverpool^7^, University of Southampton^8^, and DLR, the German Aerospace Center^9^) have been investigating the tremendous possibilities offered by Augmented Reality (AR) methods in order to further improve pilots’ safety and their performances. However, visual Augmentations must be carefully designed to optimize their beneficial effects on human performances while preventing the occurrence of detrimental effects. In this study, we examined whether deck-landing decision-making, involving a system composed of a helicopter with a bounded action capabilities and a light, 150 m-long ship of the frigate type tossed by the sea, can be improved using a visual Augmentation (A frigate is a warship whose typical length is about 150 m (Constellation class in the US, or FREMM in France) often equipped with a landing site at the stern. These ships generally have larger heave displacements due to greater sensitivity to sea waves than heavier helicopter carriers such as NATO's LHDs (Landing Helicopter Dock) (Wasp class in the United States, or Mistral in France) for example. They are traditionally used for convoy escort.). The augmentation was designed so as to take the helicopter's action capabilities into account in order to maintain the velocity at touchdown within safe limits when landing on a moving ship. Action capability is a rich and central concept in the field of ecological psychology, which is linked to affordance theory^10^, referring to Gibson’s view in the field of perception and action, according to which, the object of perception are possibilities for action. What actions are afforded to an agent in a given situation emerges from the agent’s (e.g., the pilot’s) relational properties with its environment (e.g., a helicopter / ship system)^11^. The actions afforded to a pilot depend here on both the relevant environmental properties (such as a helicopter’s ability to generate lift, its relative altitude with respect to the deck), and the relevant pilot’s properties (such as injuries preventing them from grasping the stick). In this study, a helicopter’s capability to generate lift, along with the helicopter—to—ship relationships (helicopter’s relative altitude and the relative phase of the deck oscillation) were manipulated in order to test the deck-landing-ability affordance. It is worth noting here that the concept of affordance covers more than just a helicopter’s capabilities. This point is critical to understanding how useful the concept of affordance can be for designing an Augmentation, which has been previously acknowledged in the field of Ecological Interface Design (EID)^12^.

### Assisting helicopter deck-landing: content and structure of the interface

The Ecological Interface Design framework (EID) might provide frugal solutions for designing visual Augmentations for helicopter pilots. EID has proved to be a means of improving levels of performance and safety in a large variety of activities (such as robot teleoperation^13^; missile and naval missile control^14,15^; military mission planning^16,17^), as well as enhancing aircraft pilots’ awareness of terrain^18,19^, their control of flight path and speed^20,21^, their monitoring of aircraft separation and conflict resolution^22^, helicopters’ ship deck landing^23^, helicopters’ obstacle avoidance^24^, and other tasks (for a review, see^25^).

According to the principles enacted by the EID framework, the affordances of the work domain determine the content and structure of an interface^12^. The method used to identify what affordances are relevant to pilots requires first identifying the goals pursued by pilots when landing a helicopter on the deck of a ship. Previous task analyses^1–4^ have shown that pilots aim to land with a sufficient level of accuracy on the target, with enough stability to prevent rollovers, and a touchdown velocity (${V}_{td}$) below a critical velocity (${V}_{crt}$ = 3 m s^−1^)^4^ which would otherwise risk damaging the structure of the helicopter^26^ and injure the rotorcraft crew^27^. It is secondly necessary to identify the resources with which helicopter pilots can achieve those goals. Decision-making processes resulting in deck-landing attempts appear to be closely linked to the vehicle’s action capabilities, much like other maneuvers involving motorized vehicles. For instance, drivers regulate their braking in reference to their braking capabilities^28,29^. In addition, the possibility of overtaking a leading car while avoiding oncoming traffic is perceived by taking the maximum velocity^30^ and the maximum acceleration of the driven car into account^31^. Drivers are even able to simultaneously perceive their crossing and braking possibilities in order to safely approach an intersection ^32^. The possibility of safely landing on a ship’s deck can be broadly compared with Fajen’s affordance model for braking when driving^28^. Braking consists in decelerating horizontally, whereas deck-landing consists in accelerating vertically. Both tasks require regulating the vehicle’s acceleration toward a target velocity so as to stop in front of an obstacle when driving and with a ${V}_{td}$ < ${V}_{crt}$ when deck-landing. Drivers control a car’s brakes with pedals, whereas pilots control a helicopter’s lift with the collective stick. A car's braking equipment (e.g., disc or shoe brakes and tires) determines the car’s deceleration, whereas a helicopter’s rotor and engine determine the helicopter’s vertical acceleration (i.e., its lift). While landing on the deck may seem different from automobile braking because the ship's deck may be tossed about by the waves of the sea, such movements of the target may also be encountered in actual driving situations. The main difference is the need for pilots to couple the vertical motions of their helicopter with the sinusoidal motion of the ship's deck in order to keep the future ${V}_{td}$ below a certain threshold value ${V}_{crt}$ by controlling the current lift of the helicopter (${L}_{current}$). This is expressed in Eq. (1), which describes the simplified kinematics of a vertically accelerating helicopter landing on a ship’s deck moving with a purely sinusoidal heave:

1 $${V}_{td}={V}_{current}+\left(\frac{{L}_{current}}{m}-g\right)\Delta t-A2\pi f\mathrm{cos}\left({\phi }_{current}+2\pi f\Delta t\right)$$

where *m* is the helicopter’s mass, *g* is the gravity set at 9.81 m.s^−2^, *A* and *f* are the ship’s oscillation amplitude and frequency, respectively, ${\phi }_{current}$ is its current phase and $\Delta t$ is the time remaining before touchdown. A helicopter’s lift capabilities depend on the helicopter model (especially on the engine’s characteristics), the load carried (e.g., the weight of the fuel, onboard passengers, etc.), and the atmospheric conditions. Equation (1) shows how pilots can control ${L}_{current}$ during the descent to deck with a safe ${V}_{td}$. Equation (1) describes a helicopter’s deck-landing-ability. The Eq. (1) will be used as a model of the affordance of deck-landing-ability in the present study. For the sake of completeness, it should be noted, however, that the affordance of deck-landing-ability may be determined by additional constraints, such as the type of control available to the pilot (e.g., a stick or a wheel) and his ability to use them (e.g., the pilot’s strength to overcome stick resistance).

**Figure 1:**
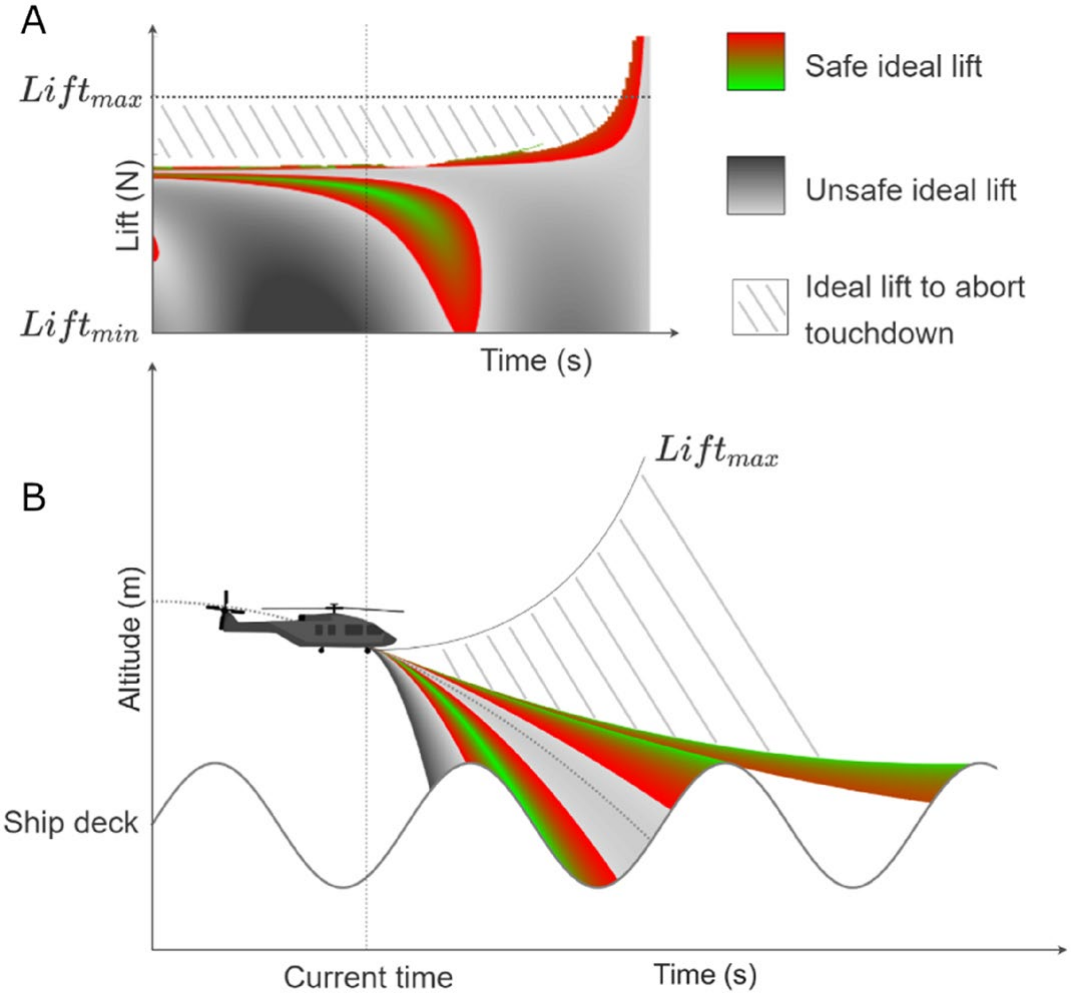
Numerical simulation of the consequences of the time-dynamics of the affordance of deck-landing-ability on (**A**) the future velocity at touchdown (${V}_{td}$) depending on the applied lift (where ${Lift}_{min}$ is the minimum lift a helicopter can reach and ${Lift}_{max}$ is the maximum lift pulling the helicopter upward), and on (**B**) the helicopter’s trajectory relative to the ship’s deck. (**A**) The applied lift can belong to three areas in the figure. When located in the white area, it rules out landing on the ship (i.e., no deck-landing should be attempted) and it is recommended to abort the deck-landing maneuver. When the lift corresponds to the colored areas, it corresponds to ideal lift values compatible with landing with ${V}_{td}$ <${V}_{crt}$ (i.e., with safe deck-landing). Green to red colors encode the distance from ${V}_{td}$ to ${V}_{crt}$. Several colored areas are presented here over time, as successive ship’s heave movements may give other possibilities for landing with ${V}_{td}$< ${V}_{crt}$. When the lift is located in gray areas, it leads to land with ${V}_{td}$ > ${V}_{crt}$ (i.e., unsafe deck-landing). White areas show lift which lead to avoid any contact with the deck. The bottom panel shows the corresponding helicopter trajectories relative to the ship deck’s vertical heave in the case of the three scenarios.

As with other affordance models^33^, the model of deck-landing-ability can help to understand both the decision-making involved in the deck-landing maneuver and the regulation of this maneuver. Following this model, pilots’ decision-making would involve deciding to deck only if they could trigger a lift resulting in an acceleration yielding to reach the deck with ${V}_{td}$ < ${V}_{crt}$. This lift could be labelled ideal, to mimic Fajen’s car’s ideal deceleration (i.e., the deceleration which, if kept constant, would make a car stop at the target) since it refers to any lift which, if kept constant, would make a helicopter reach the deck with a safe relative velocity ${V}_{td}$ < ${V}_{crt}$. As long as this ideal lift is within the helicopter’s range of lift capabilities (i.e., ideal lift < maximum lift), then safe deck-landing will be possible and pilots can trigger the deck-landing maneuver. Following again this model, pilots would have to regulate the lift to nullify the difference between the helicopter’s current and ideal lifts before the ideal lift exceeds the maximum lift. As can be seen in Fig. 1, safe deck-landing (the colored area) varies with time and depends on the helicopter’s range of lift capabilities. This means that to complete a safe deck-landing maneuver, pilots must apply the appropriate lift at the appropriate moment in time.

**Figure 2:**
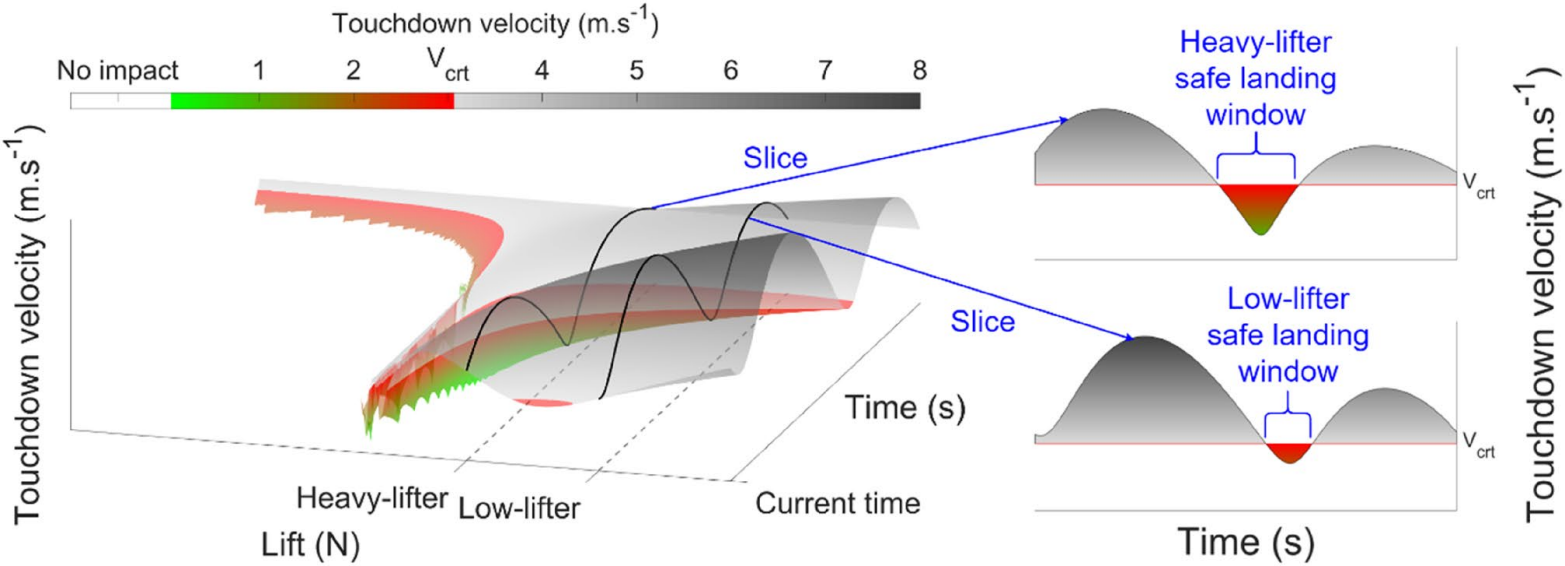
Relationship between the 3-D view of the deck-landing-ability landscape and the design of the visual Augmentation. The 3-D landscape confirms that ${V}_{td}$ changes with time as a function of the ship’s heave movements, but that it also depends on the lift applied to deck. When freezing the lift capabilities at a single ${L}_{attempt}$ value during the descent (using either Heavy-Lifter or Low-Lifter helicopters), slices can be extracted from the 3-D landscape and presented in a 2-D visual Augmentation. The 2-D visual Augmentation makes visible windows for safe deck-landing as colored valleys, during which deck-landing should be triggered so that landing can be attempted with ${V}_{td}$ < ${V}_{crt}$. The red horizontal line stands for the ${V}_{crt}$.

As this affordance-based model for deck-landing-ability is a new formalization, it was proposed in the present study to start testing its relevance to understand participants' decision-making to deck. We therefore designed a judgment task in which participants could not regulate the helicopter’s lift but were allowed to trigger a single lift value ${L}_{attempt}$ by pressing a button if and when they judged the deck-landing to be possible. First, this single lift value had implications for the experimental task. It raised the question as to whether or not participants judged the deck-landing to be possible if ${L}_{attempt}$ was to be applied to the helicopter. In our judgment task, the ship underwent a heave motion that made the affordance of deck-landing-ability dynamical, thus preserving an essential feature of the real world. The possibility of deck-landing appeared or vanished throughout a trial, as can be seen in Fig. 1. Participants therefore had to continuously distinguish between safe and unsafe deck-landing windows in order to attempt or avoid deck-landing. Second, the single lift value had implications for the visual Augmentation design. Figure 2 shows how these safe deck-landing windows can be extracted from the landscape of possibilities presented in Fig. 1 in the case of triggering a given, single, ${L}_{attempt}$. Figure 2 is a 3-D view of the previous Fig. 1 in which ${V}_{td}$ is shown in relief in order to highlight the dynamics of safe deck-landing windows, during which ${V}_{td}$ < ${V}_{crt}$. The 3-D landscape clearly shows that safe windows (colored areas) occur during valleys interspersed with longer times during which ${V}_{td}$ will be greater than ${V}_{crt}$ (gray peaks). The width and depth of these safe deck-landing windows reflect their duration and the minimum value of ${V}_{td}$ , respectively, if deck-landing had to be triggered with a single ${L}_{attempt}$ (on the horizontal axis). Using a single ${L}_{attempt}$ value is equivalent to extracting a slice along the temporal axis of the 3-D landscape. Figure 2 shows that two slices extracted from the same 3D landscape but with different ${L}_{attempt}$ values would have different widths and depths. We termed these two different ${L}_{attempt}$ “Heavy lifter” and “Low-Lifter”, with reference to the distinction in the lift capabilities (i.e., the aerodynamic force exerted by the air on the rotor) between two virtual helicopters. This illustrates thus the influence of size, engine and available payload of helicopters designed for different missions on the envelope of static and dynamic flight variables such as maximum airspeeds, accelerations, load factors, etc. In sum, different ${L}_{attempt}$, imply different lift capabilities of “Heavy lifter” and “Low-Lifter” helicopters. Indeed, it is common to manipulate vehicles' capabilities to test affordance models^28,30–32^. Decision-making outcomes are expected to follow changes in affordance. In the case of deck-landing-ability, we expected participants to trigger the landing process significantly more frequently within safe deck-landing windows, or otherwise to take the decision to abort the deck-landing maneuver.

Since Warren’s seminal work on affordance perception^34,35^, affordances have been expressed as dimensionless quantities denoted $\pi$. Expressed this way, affordances for multiple individuals in multiple situations can be compared, despite differences between individuals or vehicles. In other words, the use of affordances in terms of dimensionless quantities can be extended to many different situations, especially ones involving various helicopter capabilities. It follows that laws for making decisions and regulating behavior based on affordance models can also be generalized. As an example, Warren^34^ identified a so-called “critical point”, a value of $\pi$ at which participants' judgments about the task are liable to shift from possible to impossible. This is because the critical point marks the affordance’s boundary, where the affordance ceases to be possible. In the case of helicopter deck-landing, $\pi$ would be the ratio between ${V}_{achieved}$, the velocity achieved at touchdown if ${L}_{attempt}$ is triggered, and the critical velocity at touchdown ($\pi$ = ${V}_{achieved}$ / ${V}_{crt}$). When $\pi$ > 1, safe deck-landing would no longer be possible and a change has to occur in participants' judgments: they will abort the landing more frequently than in situations where $\pi$ < 1, despite the existence of any differences in the helicopter’s lift capabilities or the configuration of the helicopter with respect to the ship.

Another singularity of the value of $\pi$ is termed the “optimal point”. This is the value of “best-fit affordances” to use Warren’s term, a point where agents preferably find themselves ready to perform the task. In the deck-landing situation, an optimal point will be reached when ${V}_{achieved}$ = ${V}_{optimal}$, the minimal velocity at touchdown that can be reached during a trial. We will refer below to this optimal value of $\pi$ as ${\pi }_{optimal}$ (${\pi }_{optimal}$ = ${V}_{optimal}$ / ${V}_{crt}$). ${\pi }_{optimal}$ is optimal in the sense that attempting to deck at the moment in time when $\pi$ = ${\pi }_{optimal}$ yields the maximum level of safety as regards to ${V}_{crt}$. Experimental conditions where ${\pi }_{optimal}$ > 1 would mean that no safe deck-landing window would ever occur during the trial.

### Assisting helicopter deck-landing: the form of the interface

The augmentation designed to improve deck-landing maneuvers with a single, preselected lift value will show all the appropriate times for attempting a landing within safe limits (i.e., with $\pi$ < 1). To maximize safety, the visual augmentation must also indicate the optimal time for landing, defined as the moment at which $\pi$ is the lowest ($\pi$ = ${\pi }_{optimal}$), which means that ${V}_{td}$ will be as far from ${V}_{crt}$ as possible at that time.

In situations where ${\pi }_{optimal}$ is small and the span of time when $\pi$ < 1 is long, pilots will dispose of a large, deep window for deck-landing safely. This would allow pilots to attempt a larger number of landings. Conversely, situations giving pilots little or no time to initiate a landing and/or in which the associated ${V}_{td}s$ are higher (i.e., close to ${V}_{crt}$) would mean that the safe deck-landing window will be shallow and thin, or maybe even absent. The visual augmentation would allow pilots to perceive that their landing opportunities are critical or inexistent. They would then abort their landing maneuvers more serenely.

**Figure 3:**
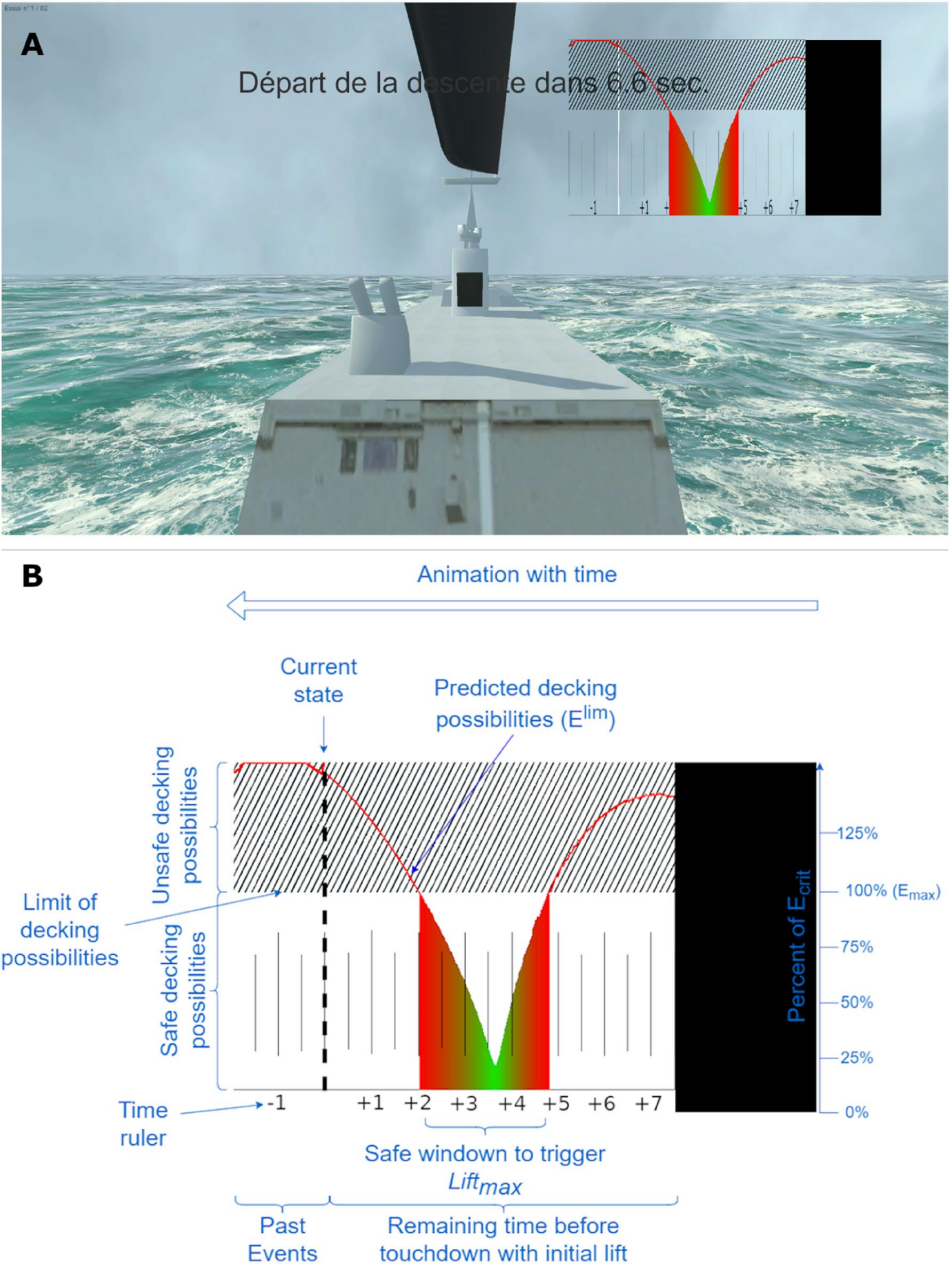
(**A**) Typical screenshot of the virtual scene during the hovering phase, including the frigate’s hangar, in front of which the ship’s deck was sitting, the top blade rotating at the top of the screen, and the Augmentation on the upper right part of the screen. The text, which was written in French, informed the participant that the initial descent was about to start in 6.6 s. (**B**) The visual Augmentation was designed to make visible the deck-landing-ability via the dynamics of the future touchdown velocity (${V}_{td}$) if ${L}_{attempt}$ was triggered. Annotations (not shown on the screen) are presented here in blue.

Slices extracted from the 3-D landscape (Fig. 2) can be used to design a 2-D augmentation displaying how the safe windows will evolve with time when the lifting capabilities are limited to a single ${L}_{attempt}$ value. Figure 3B shows the visual Augmentation designed on the basis of these slices. The presence of a small colored valley therefore enables pilots to detect the arrival in the near future of a safe deck-landing window, during which $\pi$ < 1 thus making it possible to trigger a given ${L}_{attempt}$ allowing safe deck-landing. In addition, the width of the valley reflects the duration of $\pi$ < 1, and its depth reflects the magnitude of ${V}_{td}$ if ${L}_{attempt}$ is applied. This display will change with time, ship heave movements, lift applied and the helicopter's rate of descent.

### Aim of the study

The aim of this experiment was to determine whether participants with no piloting experience perceived with the naked eye the affordance of deck-landing-ability (defining whether or not it is possible to land safely on the deck of a ship) and whether they could benefit from an ecologically designed visual augmentation assisting them to safely decide whether to attempt or abort the ship deck-landing maneuver. A simulated view of a helicopter pilot deck-landing on a frigate from hovering to touchdown was presented on a laptop screen. A visual Augmentation was designed to show the current and future deck-landing-ability during the course of a helicopter approach, taking the maximum lifting capabilities of the piloted virtual helicopter into account. The perception of deck-landing-ability was tested by manipulating the initial configuration of the helicopter/frigate system, constituting a between-trial variable and the helicopter’s action capabilities (${L}_{attempt}$) constituting a between-group variable. We examined not only whether participants were able to perceive their deck-landing-ability but also whether the visual Augmentation improved the safety of their deck-landing decisions. Lastly, it was proposed to assess how the Augmentation improved the participants’ perception of the affordance of deck-landing-ability.

## Methods

### Participants

Thirteen males and sixteen females volunteered to take part in the experiment. Nine of them received course credits. Participants' mean age was 24.4 ± 3.5 years and all had normal or corrected-to-normal vision. None of them reported having acquired any experience of aircraft piloting of any kind. The participants recruited were students at the ONERA center (Salon-de-Provence, France) and the Etienne Jules MAREY Institute of Movement Science (Marseille, France) who had responded to a volunteer search advertisement. To be eligible, participants had to be right-handed, not be regular video game players, have normal or corrected-to-normal vision, and have no experience in aircraft piloting. The sample size was determined in line with^36^ and was therefore taken to be a representative sample of young adults who were healthy but novices in the field of helicopter piloting. Participants were not informed about the aim of this study, but they were informed about the experimental procedure and signed a consent form in keeping with the requirements of the Helsinki Declaration. The experiments were performed in line with the French Public Health and APA Ethical Codes. The laptop keyboard was rigorously disinfected before each individual trial, and it was checked whether the participants were wearing masks to deal with the Covid-19 epidemic. The experiments were run from January to March 2021 at the Department of Information Processing and Systems at ONERA (Salon-de-Provence, France) and the Etienne Jules MAREY Institute of Movement Science (Marseille, France).

### Apparatus

Participants sat in front of a laptop (Intel CoreTM i7-9750H CPU @2.60 GHz with 8Go RAM, NVidia GeForce GTX 1050 GPU) operated by Windows 10 64-bit (Microsoft, Seattle, Washington, USA) and were immersed on the screen (35.4 × 19.9 cm, 1920 × 1080 px) on board a virtual NATO Frigate Helicopter. Pre-programmed rate of descent of the virtual helicopter could be changed by pressing keyboard keys to attempt or abort deck-landing maneuvers onto the deck of a Lafayette-type frigate. Both the flight dynamics of the virtual helicopter and the ship were computed online based on Eq. 1. The visual scene was rendered on the laptop screen with a Unity3D engine (2020.3.2.f1, Unity Technologies ApS, Unity 3D.com).

### Visual scene

The visual scene mimicked scenes which would be observed by a helicopter pilot landing on the deck of a Lafayette-type frigate, except that the helicopter cockpit was not visible on the screen (Fig. 3A). On the upper part of the screen, participants could see the top blades rotating while the hangar and the deck of the frigate completely occupied most of the screen in the foreground. The values of the amplitude *A* and the frequency *f* of the ship’s heave (cf. Equation 1) were always held constant at 2.5 m and 0.14 Hz, respectively. This produced a sinusoidal ship heave movement in the range of those observed under the moderate sea conditions^23^, rendered here by the Crest Ocean System^37^. A cloudy sky featured in the background.

An animated display was available in Augmentation conditions to make the deck-landing-ability visible on the upper right portion of the screen, as in a head-up display (HUD). The interface components are presented in Fig. 3B. The dynamics of the touchdown velocity ${V}_{td}$ was drawn by a curve expressed in normalized units relative to the critical touchdown velocity ${V}_{crt}$, moving from right to left with time. The critical velocity ${V}_{crt}$ was depicted in a plain horizontal black line. Any future touchdown velocity ${V}_{td}$ below this line meant that safe deck-landing was safe, whereas those located above the line in the black hatched part corresponded to unsafe deck-landing. To show up the safe deck-landing possibilities, the area under the curve at times when ${V}_{td}$ < ${V}_{crt}$ were colored with colors ranging from pure green (${V}_{td}$ = 0) to red (${V}_{td}$ = ${V}_{crt}$), in line with^38^. A horizontal ruler displayed on the abscissa at the bottom of the display gave the time series in seconds. The dotted vertical line indicates the current time. Its intersection with the curve indicated the ${V}_{td}$ which would be reached if ${L}_{attempt}$ was triggered immediately. The left part of the ${V}_{td}$ curve corresponds to the past touchdown velocity, and the right part to the near future touchdown opportunity. The left edge of the black area stands for the end of the trial. The colored curve is periodically deformed with time, depending on the ship heave movements, and includes emerging and vanishing safe windows for triggering ${L}_{attempt}$. As the helicopter moves toward the ship, the colored curve and the black area move from right to left until the black area reaches the vertical line defining the current time.

## Procedure

### Task

Upon their arrival at the Lab, participants were informed that their task consisted in answering a single question: Press the "Yes" button if and when you judge that your lift capability will enable you to land safely on the ship’s deck or press the "No" button as soon as you perceive that it will never be possible to deck safely". They were informed about the trial procedure and how the Augmentation worked.

### Protocol

Prior to the experiment, during the calibration phase, participants performed practice trials in order to calibrate themselves with the simulation dynamics. During the experimental phase, their responses were monitored. Immediately after the end of the experimental phase, participants completed a Modified Cooper-Harper for Unmanned Vehicle Displays (MCH-UVD) questionnaire.

### Unfolding of trials

**Figure 4:**
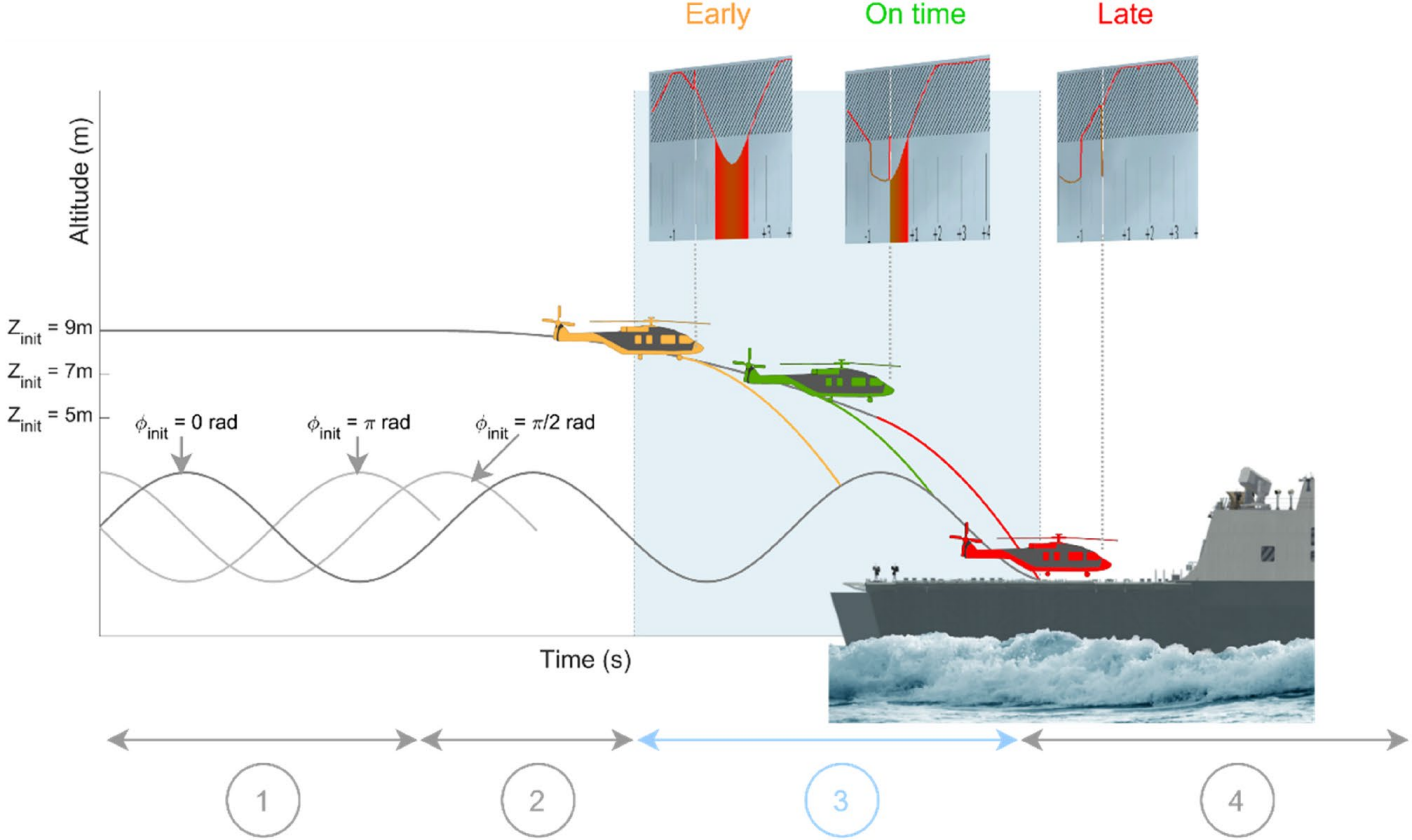
Unfolding of trials. (1) the helicopter hovered in a stationary position in Earth reference at an initial altitude ${Z}_{init}$. The countdown indicated the remaining time in this stationary position. (2) The helicopter slowly decreased altitude (lift is reduced to 97,119 N). A second countdown was then presented, showing, how much time was left before participants were allowed to press either the "yes" or "no" button. (3) The second countdown disappeared when it reached 0 s. and participants were then allowed to either attempt the deck-landing maneuver by pressing "yes", or abort it by pressing "no". (4) If no response occurred, the helicopter reached the deck and participants were given some feedback information to encourage them to respond in the subsequent trials. Grey arrows show the phases during which participants could not press the "yes" or "no" buttons (they were instructed not to respond during these phases, and any responses occurring during these phases were not taken into account). The light blue arrow shows the time interval during which participants were expected to produce a response.

The unfolding of trials (Fig. 4) was similar during both the calibration and experimental phases. Trials always started with the helicopter hovering in a stationary position for 14 s in Earth reference above the center of the ship’s deck. This period corresponded to two cycles of the ship’s heave and facilitated the pickup of the ship’s heave amplitude, frequency and phase. During this period, the first countdown informed participants how much time remained for them to stay in the hovering mode. When this first countdown was over, the helicopter started to descend with a lift set at 97.119 N (99% of *m* ∗ *g*) and a second countdown started, informing participants when they would be allowed to respond. Once the second countdown was over, the helicopter would continue to descend at the same rate until the participants pressed a key. If they pressed the "Yes" key, the helicopter’s rate of descent changed according to a constant ${L}_{attempt}$ value (90,000 N for Heavy Lifters and 80,000 N for Low Lifters) until reaching the deck. If the participants pressed the "No" key, the deck-landing maneuver was aborted, and a new trial started. If neither key was pressed, the helicopter reached the deck with its initial rate of descent.

### Feedback

Participants were provided with feedback information at each trial during the calibration and experimental phases via short text messages. The first message indicated whether or not the decision to attempt or abort the deck-landing maneuver was appropriate in view of their deck-landing possibilities. The second message, which occurred whenever a deck-landing maneuver was correctly attempted, indicated whether the timing of the deck-landing attempt was early, on time or late with respect to their deck-landing possibilities. After each trial in the calibration phase, the value of the touchdown velocity was provided in addition to the second message in order to favor the calibration.

### Independent variables

The calibration phase comprised 18 trials. Three combinations of initial altitudes (${Z}_{init}$) and phases (${\phi }_{init}$) were used ([${Z}_{init}$; ${\phi }_{init}$] ∈ {[9 m;0 rad],[5 m; $\pi$ rad],[7 m; $\pi$ /2 rad]}).

During the experiment phase, the virtual helicopter’s attempt lift (${L}_{attempt}$), serving as a between-group variable with two levels (80,000 N and 90,000 N), was manipulated. For this purpose, participants were randomly divided into two mixed gender groups. 15 participants were assigned to a Low-Lifter (LL, ${L}_{attempt}$ = 80,000 N) and the other 14 to a Heavy-Lifter (HL, ${L}_{attempt}$ = 90,000 N) virtual helicopter. These values were chosen to induce differences in the vertical rates of descent between HL and LL groups, which in turn would induce differences in their deck-landing-ability.

We also manipulated the deck-landing-ability affordance at the start of each trial as a between-trial variable, summarized by 2 ∗ 8 ${\pi }_{optimal}$ quantities ranging from 0.22 to 1.07 for the LL and from 0.03 to 1.11 for the HL group. The ${\pi }_{optimal}$ values used here resulted from varying 8 pairs of parameters in the initial state of the helicopter/frigate system: the initial altitudes (${Z}_{init}$ = 5 m, 7 m and 9 m) and the initial phases of the frigate (${\phi }_{init}$ = 0 rad, $\pi$ /2 rad and $\pi$ rad). Since the pair [${Z}_{init}$ = 5 m: ${\phi }_{init}$ = 0 rad] pair did not give a long enough descent phase (zones 2 and 3 in Fig. 4) for participants to have enough time to produce a response, this pair was not tested in this experiment. This yielded 8 conditions of deck-landing-ability for each of the two virtual helicopters.

Lastly, the availability of the Augmentation as a between-trial variable (2 modalities: Control without Augmentation and Augmentation) was manipulated. The 8 ${\pi }_{optimal}$ conditions were repeated 4 times in each of the 2 modalities of Augmentation availability and presented in random order, resulting in a total number of 64 trials.

### Data analysis

We eliminated from the analysis all trials in which participants forgot to press a key (2.3% of the trials). The trials in which participants pressed two different keys were analyzed by analyzing the last key pressed (4.2% of the trials).

*Assessing the benefits of the Augmentation:* the benefits of the Augmentation were first analyzed in terms of participants’ decision-making ability to distinguish between safe and unsafe deck-landing possibilities. In each trial, we either retrieved ${V}_{td}^{attempt}$, the touchdown velocity resulting from attempted deck-landing, or simulated a posteriori ${V}_{td}^{abort}$, the touchdown velocity that would have been reached if the maneuver had been pursued with ${L}_{attempt}$ at the moment when the maneuver was aborted. The benefits of the Augmentation were analyzed secondly using the Modified Cooper-Harper for Unmanned Vehicle Displays (MCH-UVD) diagnostic tool^39^ to determine how useful the Augmentation was to the participants. The questionnaire was completed by participants after the experiments. Individual Modified Cooper-Harper ratings were converted into Z-scores before the analysis.

*Assessing deck-landing-ability perception:* the benefits of the Augmentation for perceiving the deck-landing-ability were analyzed by studying the effects of $\pi$-conditions on the frequency and timing of participants’ deck-landing attempts. Traditionally, analyzing the perception of an affordance involves studying the parameters of a sigmoid function^40^ that fits the response frequency expressed in either an extrinsic or an intrinsic unit $\pi$^30,31,35^. This analysis is possible as long as there is only one value of $\pi$ per experimental condition, which makes for a sufficiently large total number of responses and a good balance between experimental conditions. In our model of the affordance of deck-landing-ability, the $\pi$-value varies during the course of trials, and the $\pi$-value analyzed depended on the moment when participants responded (by attempting or aborting deck-landing). The number of individual responses per $\pi$ value can therefore change until it becomes unrepresentative of the actual cognitive process (i.e., an error occurring among only a few responses at a given value of $\pi$ would have too high a cost). We therefore decided to run Anovas on the response frequency instead of analyzing parameters of the sigmoid functions, a valid alternative that has been previously applied in studies on affordances^34^.

*Assessing perception of the optimal timing of deck-landing-ability:* the benefits of the augmentation in terms of finding the optimal timing for attempting a deck-landing maneuver were examined by computing the error between the optimal timing featuring in safe deck-landing windows and the moment at which deck-landing was attempted. Optimal timing was defined as the moment at which the deck-landing attempt would result in a minimum ${V}_{td}$, that is the moment at which the safe deck-landing window was the deepest in Fig. 3A.

## Results

### Attempt rates

Deck-landing attempts were first examined in order to quantify the benefits of the Augmentation under safe and unsafe deck-landing conditions. A 3-way mixed model ANOVA (2 groups [LL, HL], 8 deck-landing-ability levels [${\pi }_{optimal}$], 2 Augmentation availabilities [Control, Augmentation]) with repeated measures on the ${\pi }_{optimal}$ was run on the frequencies of deck-landing attempts. The ANOVA showed the existence of significant main effects of both the availability of Augmentation (F(1, 27) = 19.36, *p* < 0.001, ${\eta }_{p}^{2}$ = 0.42) and the deck-landing possibilities $\pi$ (F(3.51, 94.70) = 76.35, *p* < 0.001, ${\eta }_{p}^{2}$ = 0.74) as well as significant group × Augmentation (F(1, 27) = 6, *p* = 0.02, ${\eta }_{p}^{2}$ = 0.18), and $\pi$×Augmentation availability interactions (F(4.17, 112.49) = 27.38, *p* < 0.001, ${\eta }_{p}^{2}$ = 0.50). A group × $\pi$×Augmentation availability interaction was also observed (F(4.17,112.49) = 2.96, *p* = 0.02, ${\eta }_{p}^{2}$ = 0.01). The Tukey HSD post-hoc test showed that the effect of deck-landing possibilities was mostly due to significant differences in deck-landing attempts between conditions where safe windows were available (average ${\pi }_{optimal}$ = 0.24, 0.61, 0.51 and 0.13) and those where they were not available (average ${\pi }_{optimal}$ = 1.05, 1.07; *p* < 0.001) or where they were small (average ${\pi }_{optimal}$ = 0.78; *p* < 0.01). These results suggest that participants' decisions to attempt or abort deck-landing depended on the availability of safe deck-landing windows during the course of a trial. The Tukey HSD posthoc tests also showed that the frequency of deck-landing attempts by the HL group was significantly reduced by the use of the Augmentation under all conditions where no safe windows were present (${\pi }_{optimal}$ = 1.11, 1.05 and 1.07; *p* < 0.001) in comparison with the Control condition. The frequency of deck-landing attempts by the LL group was almost significantly reduced by the Augmentation when ${\pi }_{optimal}$ = 1.07 (*p* = 0.053) and was significantly reduced in the unsafe condition where ${\pi }_{optimal}$ = 1.05 (*p* < 0.01).

**Figure 5:**
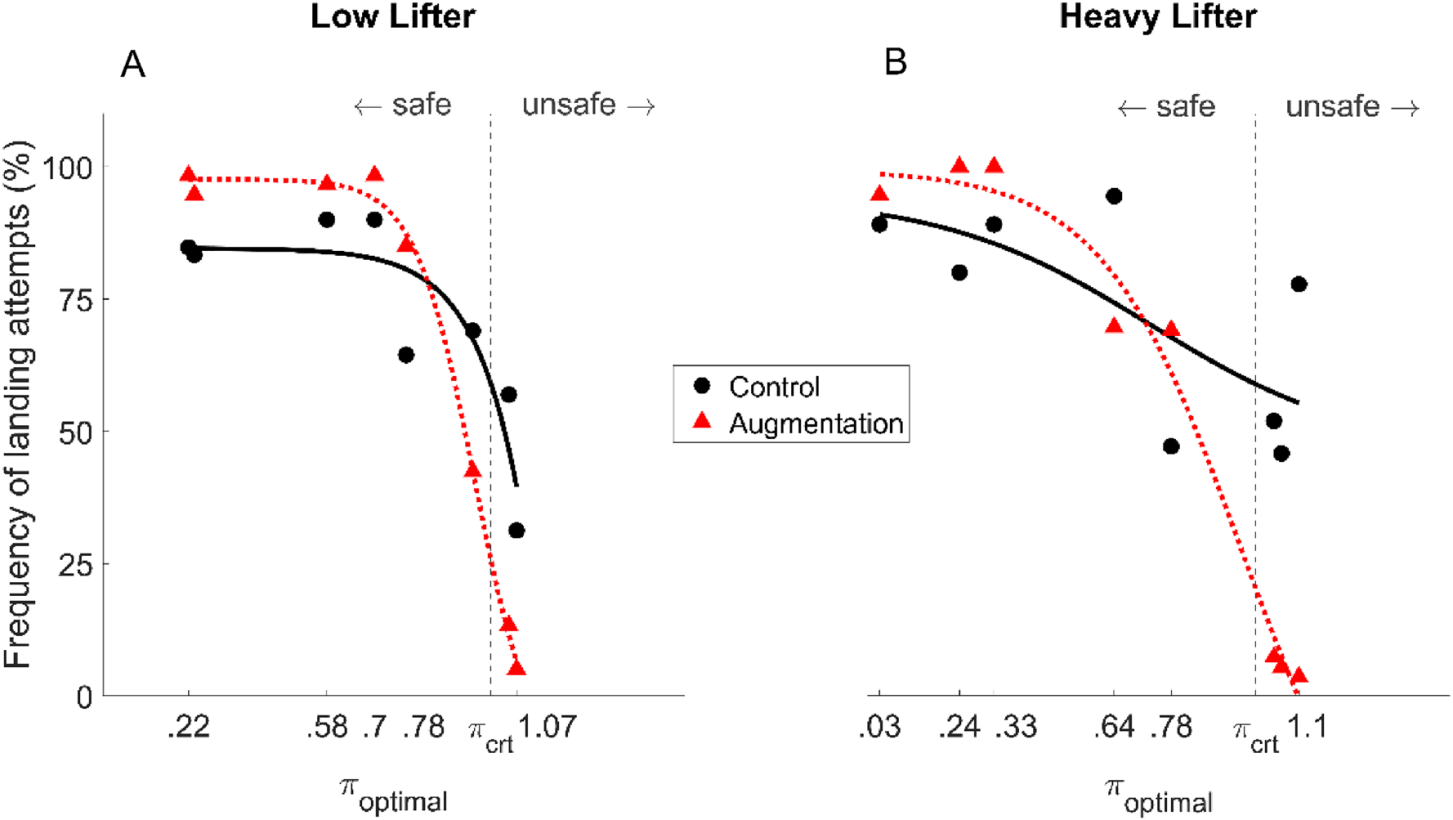
Inter-individual mean frequency of attempted deck-landing maneuvers expressed as a function of ${\pi }_{optimal}$ for Control (**A**, **B**) and Augmentation conditions (**C**, **D**) and for LL (**A**–**C**) and HL (**B**–**D**) groups. The red curve depicts the mean sigmoid function fitted on inter-individual mean frequency.

To summarize, the Augmentation was found to be most beneficial during unsafe deck-landing windows, where it decreased the number of deck-landing attempts from about 40% to less than 10% from Control to Augmentation conditions (Fig. 5). The Augmentation is also beneficial during safe deck-landing windows, where it maximizes the safety of deck-landing attempts.

### Touchdown velocities

**Figure 6:**
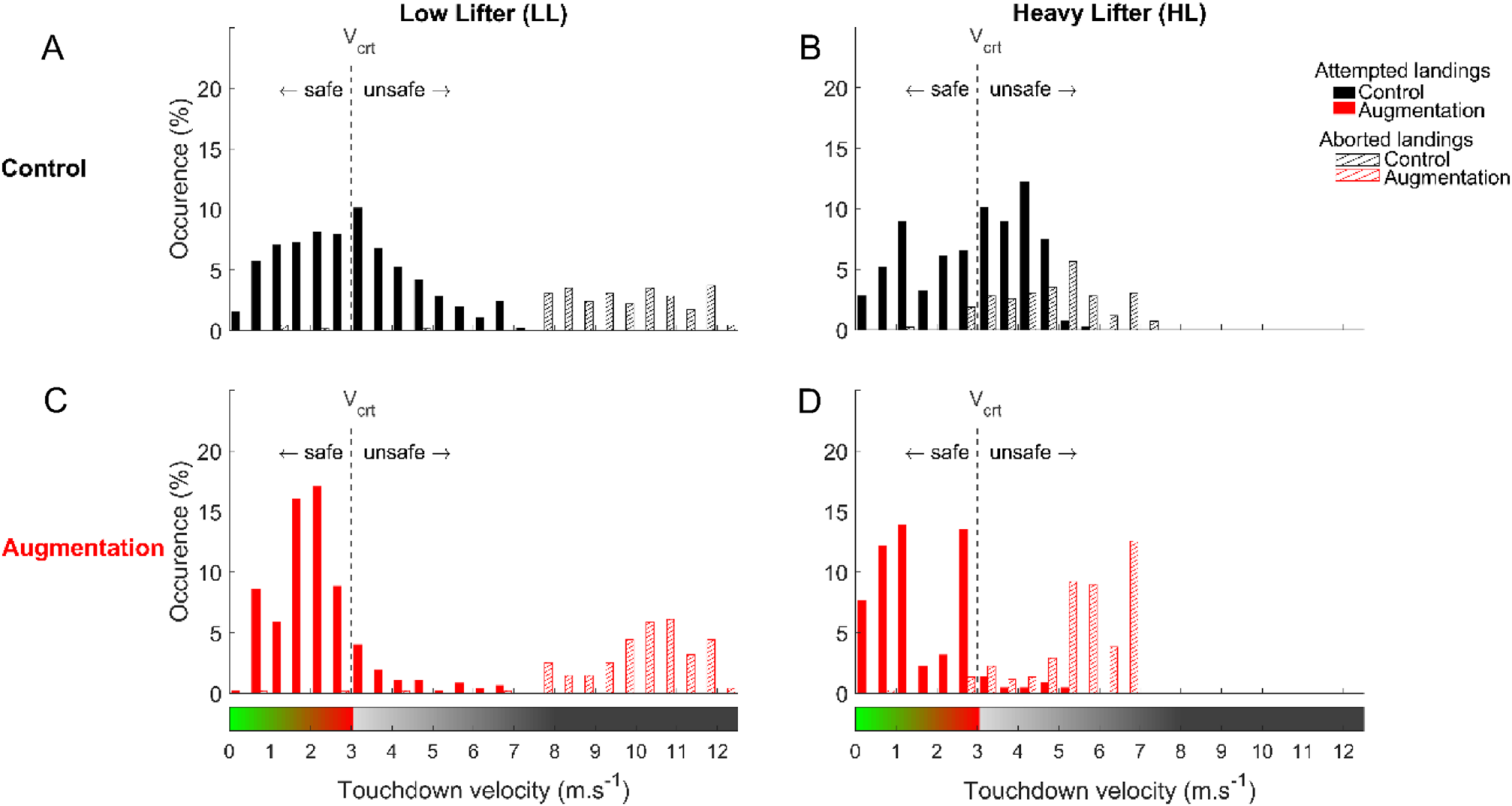
Distributions of touchdown velocity resulting from attempting deck-landing (${V}_{td}^{attempt}$) and predicted when aborting deck-landing (${V}_{td}^{abort}$) for Control (**A**, **B**) and Augmentation conditions (**C**, **D**) and for LL (**A**–**C**) and HL (**B**–**D**) groups. The red dotted vertical line depicts ${V}_{crt}$ and separates the safe touchdown velocities from unsafe ones.

Touchdown velocity was also analyzed to investigate whether the Augmentation reduced the number of unsafe deck-landing attempts (i.e., those occurring above ${V}_{crt}$) with respect to the Control, non-augmented condition. In the Control condition, the distribution of touchdown velocities resulting from attempted deck-landing (${V}_{td}^{attempt}$) represented in the form of filled bars in Fig. 6, was roughly centered around ${V}_{crt}$. Participants therefore attempted to deck despite unsafe deck-landing conditions, with ${V}_{td}^{attempt}$ > ${V}_{crt}$, in more than one-third of the trials (36.09 ± 15.60 and 41.50 ± 16.28% in the case of participants in the LL and HL groups, respectively). In the Augmentation condition, the distribution of ${V}_{td}^{attempt}$ shifted below ${V}_{crt}$. Participants therefore made few attempts resulting in unsafe deck-landing (10.80 ± 13.37 and 5.28 ± 4.14% of trials in the case of LL and HL groups, respectively). A 2-way mixed-model ANOVA (2 groups [LL,HL], 2 Augmentation availabilities [Control, Augmentation]) with repeated measures on the availability of Augmentation run on the frequency of ${V}_{td}^{attempt}$ > ${V}_{crt}$ supported these findings by showing that the Augmentation significantly reduced the number of unsafe deck-landing attempts in comparison with the Control condition (F(1, 27) = 112.35, *p* = 0.04, ${\eta }_{p}^{2}$ = 0.81), independently of the group.

We also wondered whether the Augmentation maximized the attempts resulting in safe deck-landing maneuvers, encouraging participants to abort deck-landing maneuvers only when they were unsafe. The distribution of touchdown velocities predicted from aborted deck-landing (${V}_{td}^{abort}$), presented in the form of hatched bars in Fig. 6, was centered well above ${V}_{crt}$ in the Control and even more so in the Augmentation condition. This suggests that participants mainly aborted deck-landing when it was unsafe and that the Augmentation strengthened this phenomenon. A 2-way ANOVA with repeated measures run on the frequency of ${V}_{td}^{abort}$ > ${V}_{crt}$ confirmed this finding by showing that the Augmentation significantly reduced the number of aborted maneuvers that would have led to a safe deck-landing in comparison with the Control condition (F(1, 27) = 8.944, *p* = 0.006, ${\eta }_{p}^{2}$ = 0.24), independently of the group.

Overall, in the Control condition, participants attempted deck-landing mainly within or near safe windows and aborted deck-landing only when it was unsafe. The Augmentation improved the safety of participants’ decision-making, since deck-landing attempts were even more densely distributed within safe windows, whereas deck-landing maneuvers were aborted around higher values of ${V}_{td}^{abort}$.

### Timing of decisions

The timing of deck-landing attempts was the third point analyzed in order to check whether participants' decision to deck was taken randomly or whether it might be related to the availability of safe deck-landing windows. A 3-way mixed model ANOVA (2 groups [LL, HL], 5 deck-landing-ability levels [${\pi }_{optimal}$], 2 Augmentation availabilities [Control, Augmentation]) with repeated measures on the ${\pi }_{optimal}$ providing safe deck-landing windows (mean ${\pi }_{optimal}$ = 0.78, 0.24, 0.61, 0.51 and 0.13) was performed on the timing of deck-landing attempts. The ANOVA shows a significant main effect of the deck-landing-ability conditions (F(1.78, 37.42) = 144.82, *p* < 0.001, ${\eta }_{p}^{2}$ = 0.87) as well as a significant deck-landing-ability × Augmentation interaction (F(4, 84) = 30.98, *p* < 0.001, ${\eta }_{p}^{2}$ = 0.60). These data suggest that the timing of participants’ attempts to deck was not random. Rather, the effects of the $\pi$-condition suggest that the timing of participants’ decisions is organized on the basis of some property of the various $\pi$-conditions, which might be the optimal time at which $\pi$ = ${\pi }_{optimal}$. According to this hypothesis, participants' decision to deck should be made in line with the optimal timing. The Tuckey HSD posthoc tests support this idea, since the effect of the $\pi$-condition on the timing of attempted deck-landing in the Control condition was observed between all the $\pi$-conditions (*p* < 0.01 with all pairs) except between conditions [5 m; $\pi$/2] and [7 m; $\pi$/2] (*p* = 0.79) and between conditions [9 m; 0] and [7 m; 0] (*p* = 0.83). These pairs of conditions showed the smallest differences in the timing when $\pi$ = ${\pi }_{optimal}$ (= 0.39 and 0.64 s between conditions [5 m; $\pi$/2] and [7 m; $\pi$/2] in groups LL and HL, respectively, and = 0.70 and 1.20 s between conditions [9 m; 0] and [7 m; 0] in groups LL and HL, respectively). All in all, these results suggest that large enough variations in optimal timing for attempting deck-landing would result in significant variations in the timing at which participants decided to trigger the landing, even in the Control condition. This might be explained by the fact that optimal timing and timing when there is a safe deck-landing window available are indeed linked to each other and were both taken into account here by the participants.

**Figure 7:**
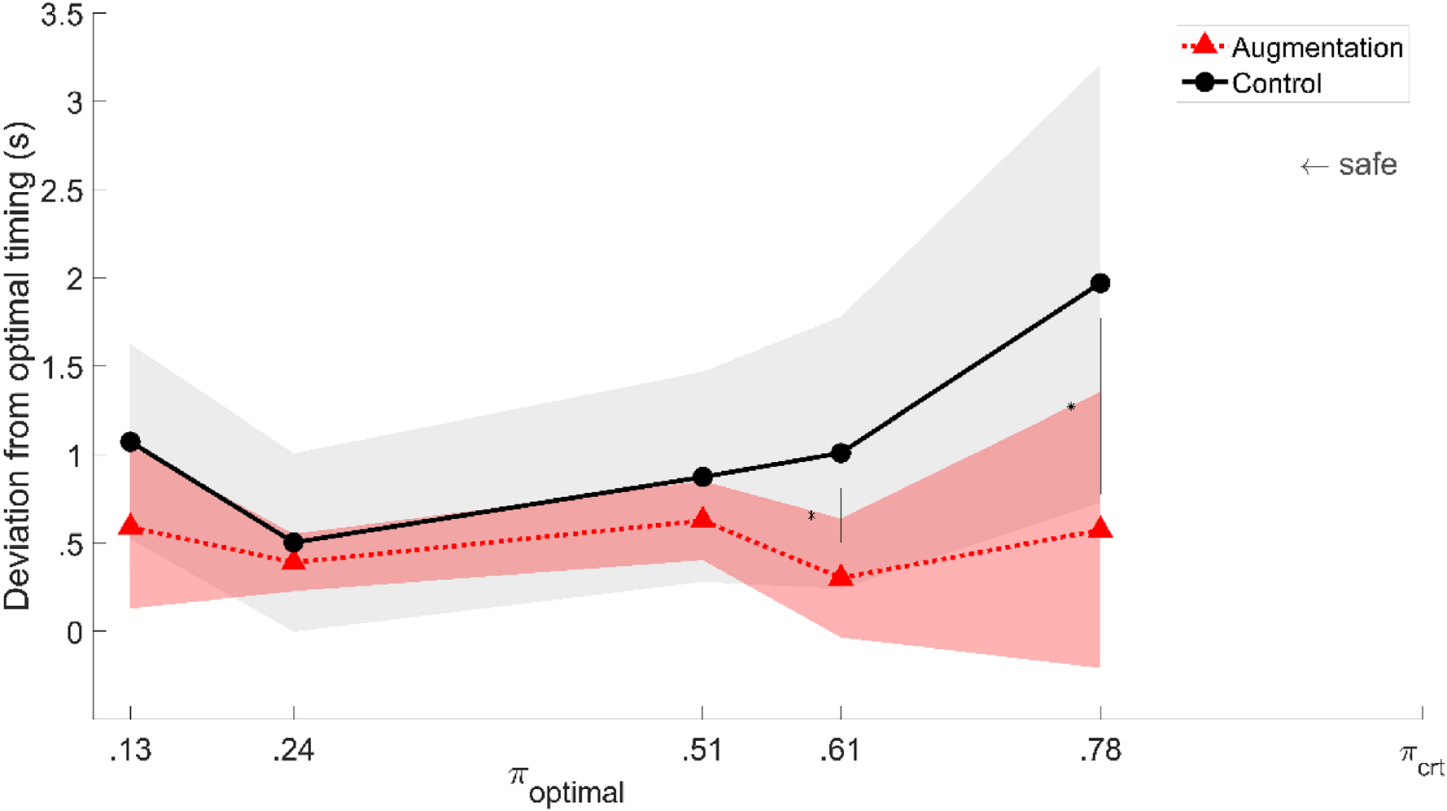
Inter-individual average timing error between the moment of the deck-landing attempt and the optimal moment during safe deck-landing windows as a function of ${\pi }_{optimal}$ in Control and Augmentation conditions (plain black and dotted red curves, respectively). Data on LL and HL groups were pooled together. The colored areas give the standard deviations of individual means.

By focusing on the magnitude of error in the timing of deck-landing attempts, we expected to obtain further insights into the benefits of the Augmentation as a means of selecting the appropriate time at which to attempt deck-landing. A 3-way mixed model ANOVA (2 groups [LL,HL], 5 deck-landing-ability levels [${\pi }_{optimal}$], 2 Augmentation availabilities [Control, Augmentation]) with repeated measures on the $\pi$-condition providing safe deck-landing windows was performed on the average individual error between the timing of deck-landing attempts and the optimal timing. The analysis showed the existence of significant effects of both the $\pi$-condition (F(1.86, 39.04) = 7.72, *p* < 0.01, ${\eta }_{p}^{2}$ = 0.27) and the use of Augmentation (F(1, 21) = 46.82, *p* < 0.001, ${\eta }_{p}^{2}$ = 0.69), with no significant main effects of the group (F(1, 21) = 2.12, *p* = 0.16, ${\eta }_{p}^{2}$ = 0.09). In addition, a $\pi$×Augmentation availability interaction was observed (F(2.30, 48.40) = 12.55, *p* < 0.001, ${\eta }_{p}^{2}$ = 0.37). The results are displayed on Fig. 7. The Tukey HSD posthoc tests showed that Augmentation significantly decreased the magnitude of timing errors in the two $\pi$-conditions where the amount of time available for attempting safe landing was the shortest (i.e., ${\pi }_{optimal}$ = 0.61 and 0.78 on average in both HL and LL groups). These results suggest that the Augmentation was a beneficial means of finding the optimal timing of deck-landing attempts.

### Landscape of deck-landing-ability

**Figure 8:**
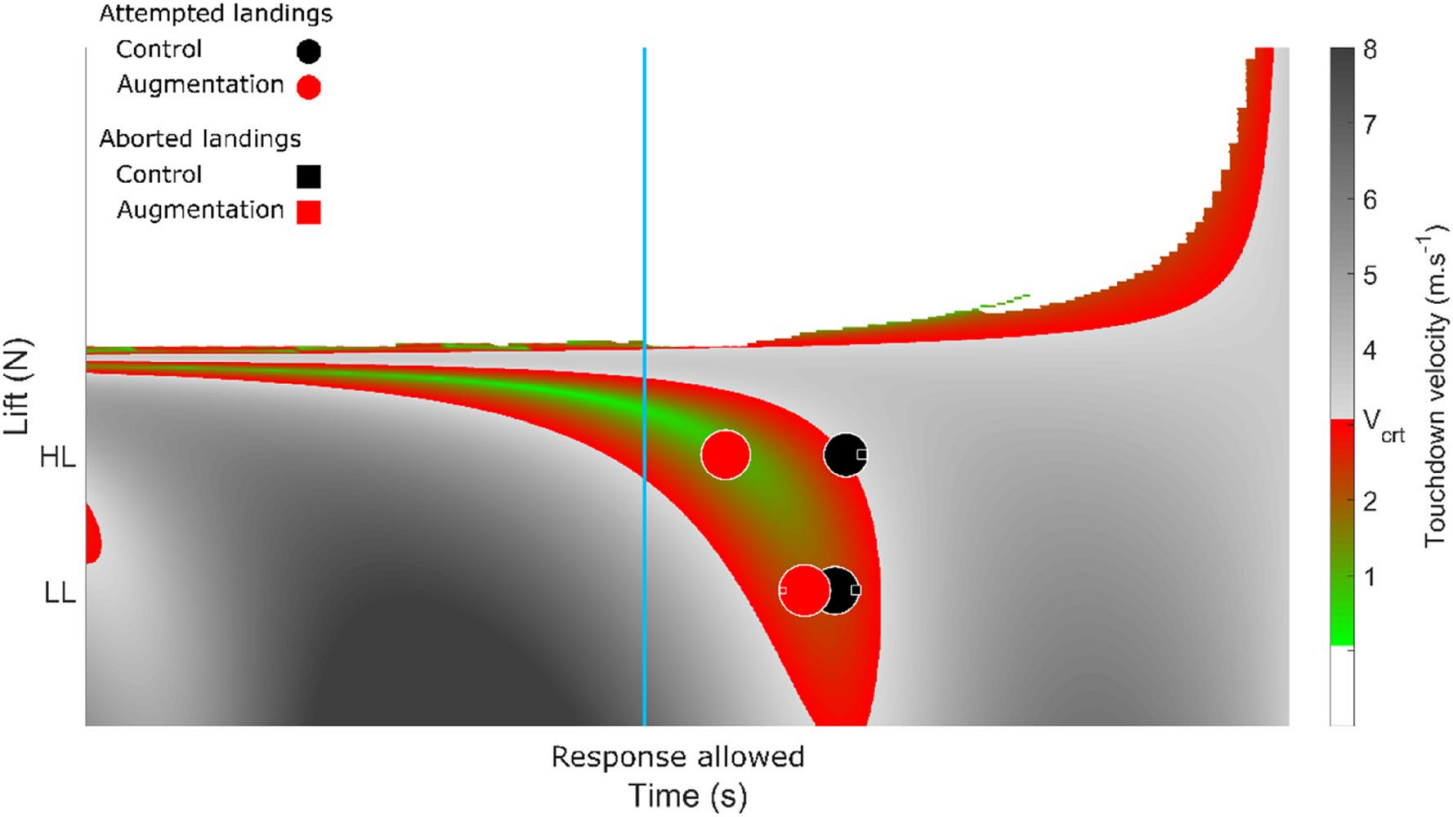
Inter-individual average frequency of attempted and aborted deck-landing (circles and squares symbols, respectively) under Control and Augmentation conditions (black and red, respectively) plotted in the condition [9 m; 0 rad] in which safe deck-landing was possible (${\pi }_{optimal}$ = 0.70 and 0.32 with groups LL and HL, respectively) over the deck-landing-ability landscape. The size of the symbols reflects the frequency of decisions. The green to red colors encode the distance from ${V}_{td}^{attempt}$ to ${V}_{crt}$, thus indicating the safety of the deck-landing attempt. Light to dark gray show unsafe deck-landing situations where ${V}_{td}$ > ${V}_{crt}$.

The nature of the decision (to attempt or abort deck-landing), its frequency, its outcome (i.e., the velocity at touchdown) and its timing can be captured together in the landscape of deck-landing-ability shown in Fig. 8 (cf. Figure 2 for explanations). The deck-landing-ability is reflected in the width of the colored thong that stands for the duration of the safe deck-landing windows, and in the green to red colors encoding the distance of ${V}_{td}$ from ${V}_{crt}$, thus showing the safety of the deck-landing attempt. The nature of the decision (attempt vs abort) is depicted in the form of symbols (circles and squares standing for attempted and aborted maneuvers, respectively), the frequency of the decision is indicated by the size of the symbol, the velocity at touchdown relative to ${V}_{crt}$ is encoded by green to red colors. Lastly, the black and red colors correspond to Control (non-augmented) and Augmentation conditions, respectively. Figure 8 shows first that the Augmentation maximized the deck-landing attempts as compared with the Control condition (98% and 100% vs. 90% and 89% attempts by the groups LL and HL, respectively). Figure 8 also makes it clear that the Augmentation brings the timing of the deck-landing attempt closer to the optimal timing than in the Control, non-augmented condition, so as to keep the velocity at touchdown below ${V}_{crt}$ (2.13 and 0.99 m.s-1 vs. 2.24 and 3.11 m.s-1 in both Low and Heavy Lifter groups).

### Subjective ratings

Lastly, the perceived usefulness of the Augmentation was analyzed in order to complete this picture of the benefits of the Augmentation. The average ratings of MCH-UVD in the Augmentation condition were equal to 2.31 ± 2.54 (i.e., almost perfect perceived usability). A bilateral t-test for independent samples (2 groups [LL, HL]) performed on the individual ratings did not bring to light any significant differences between the groups HL and LL. In short, all the participants perceived the Augmentation as facilitating the decision-making process. These results are in line with the benefits of the Augmentation observed here on participants’ performances.

## Discussion

In this study, it was first proposed to model and investigate the perception of the affordance of deck-landing-ability, defined as the possibility of landing safely on the deck of a moving ship, based on the available lift potential. Moreover, we designed a visual Augmentation which intended to improve the decision to either attempt or to abort the deck-landing maneuver by presenting with the current and future deck-landing-ability during the ongoing approach. The deck-landing-ability presented on a laptop simulator was manipulated in order to investigate not only whether participants succeeded in deck-landing safely under non-augmented conditions but also whether the visual Augmentation actually improved their decision-making performances.

### Naked eye perception of deck-landing-ability

The three main results obtained in this study suggest that participants perceived with the naked eye only roughly the affordance of deck-landing-ability as expressed in Eq. 1. Participants' decision to trigger or abort the deck-landing maneuvers and the timing at which they triggered the deck-landing were both consistent with our predictions and depended significantly on $\pi$. The distribution of aborted and attempted landings with reference to ${V}_{crt}$ confirmed this hypothesis: the ${V}_{td}^{attempt}$ values were distributed around touchdown velocities below ${V}_{crt}$ (i.e., those in the safe deck-landing window), whereas the ${V}_{td}^{abort}$ values were distributed around touchdown velocities above ${V}_{crt}$ (i.e., those in the unsafe deck-landing window). Participants therefore tended, to take into account at least to some extent the relationship between their future touchdown velocities and the value of ${V}_{crt}$ when deciding whether or not to attempt deck-landing. Their perception of the affordance of deck-landing-ability was not very accurate, however. Participants triggered some of their landings outside the safe deck-landing window (cf. Figure 6, where it can be seen that the distribution of attempted landings shifted towards higher ${V}_{crt}$ values) and their deck-landing attempts plateaued around 50% (instead of ideally, 0%) when safe deck-landing was impossible (cf. Figure 5, which shows the conditions where ${\pi }_{optimal}$ > 1). The first explanation for this situation might be that participants may have overestimated ${V}_{crt}$. Another possibility might be that participants did not perceive the other component of the ratio determining the deck-landing-ability, namely ${V}_{achieved}$. This could happen if any of the variables in Eq. 1 could not be accurately detected. In particular, ${V}_{achieved}$ was partly determined by the future state of the ship at helicopter touchdown. The future state of the ship can be estimated based on the current state^41^. Under realistic conditions, expert pilots combined the vertical displacements of their helicopter with the heave motion of the ship prior to deck-landing. This coupling would enable them to explore the future swell state of the ship so as to keep the energy at impact below a certain threshold value^42^. We therefore hypothesized that this exploratory coupling would make the affordance of deck-landing-ability more salient and thus facilitate its perception. As the current study does not make it possible to actively control the helicopter’s vertical movements, future experiments are now required in which participants are able to perform this exploratory coupling process, which would improve their perception of the situation (see Gibson’s experiments on active touch^43^). Lastly, a lack of information while hovering during the observation phase might provide a third explanatory hypothesis. Pritchard et al.^3^ have studied pilots gazing at the following three landmarks during the hovering and landing phases: one located on the flight deck at the intersection between the lineup line and the hangar, one just above that point in the top corner of the hangar roof, and one on the horizon. In the present study, only the last two landmarks were continuously available to the participants, since the first one was available only after the helicopter had started its descent towards the deck.

### Augmentation of the affordance of deck-landing-ability

The present ecologically designed Augmentation improved the safety of participants with no piloting experience. Our analyses of ${V}_{td}^{attempt}$ showed that the Augmentation significantly reduced the number of unsafe attempted deck-landing maneuvers (i.e., those endangering the helicopter’s structure and participants’ physical state) in comparison with the non-augmented, Control conditions. This is a great step forward in the prevention of deck-landing fatalities. Likewise, our analyses of ${V}_{td}^{abort}$ showed that the Augmentation significantly reduced the number of aborted maneuvers when safe deck-landing windows were available. This method could also save lives because it prevents the hover phase from lasting, thus preventing an increase in workload and frustrations^4^ and the resulting occurrence of deck-landing attempts outside safe landing windows. Our analysis of individual MCH-UVD ratings also showed that the participants perceived the Augmentation as a means of facilitating the decision-making process. We then attempted to elucidate how the Augmentation improved participants' decision-making. We first established that the benefits of the Augmentation originated both from the fact that it enabled participants to make a clear-cut distinction between safe and unsafe deck-landing windows. It decreased the number of deck-landing attempts corresponding to unsafe deck-landing conditions and maximized those corresponding to safe deck-landing conditions. Secondly, we observed that the Augmentation enabled participants to time their deck-landing attempts optimally. These benefits are apparently greater than those obtained using the visual Augmentation method developed by Morice et al.^23^, who did not find any significant improvement in ${V}_{td}$. More generally speaking, the results obtained in the present study support the idea that understanding the agent/environment system in terms of what it affords is a fruitful approach to designing meaningful Augmentations. We readily acknowledge the fact that the aforementioned affordance of deck-landing-ability includes more than just the involved energy constraints and that the use of several levels of abstraction may not yet be fully integrated. However, the present model of the affordance of deck-landing-ability shows the advantages of affordance-based control models developed on similar lines to those presented by Fajen in the context of car braking^28,29^ and locomotion^33^. The latter models included the behavioral regulation aspects, which seem to be particularly useful for designing an interface improving the performance of goal-based visually controlled tasks. We therefore believe that interface design should be grounded on a solid theoretical basis, while upholding the meaningfulness of perception as part of an agent/environment relationship^44^. Only in this way will designers be able to develop interface displays that convey this meaning to the operator.

The form of the present Augmentation differs from projective displays such as those developed by Kruit^45^ for assisting rally drivers by overlaying lines on the road using an HUD to regulate the cornering. In our own case, the use of an HUD is justified by the fact that it minimizes the amount of visual switching required between inside and outside the cockpit, thus decreasing pilots’ workload. The form of our interface also differs from previous EID applications such as DURESS^46^, in which the interface took the form of geometric shapes standing for relevant aspects of a nuclear power plant. In the present application, the deck-landing-ability is depicted in a more graphic way, as in the force ratio and force ratio trend displays developed for military control systems by Hall et al.^47^. Despite these differences, the form of the present interface allows the emergence of several relevant properties. For example, the closing rate between the black area indicating the end of the trial and the white vertical line indicating the current time makes it possible to visualize the closing rate between the helicopter and the deck. Likewise, the distance between these components indicates the time-to-contact with the deck. The emergence of these properties is possible because the highly structured information brings to light the affordances at work, as in the DURESS or Kruit interfaces.

There exist a whole set of clues suggesting that the present visual Augmentation may also be beneficial to experienced helicopter pilots. The present study involved participants with no prior skills in aeronautics, as the samples tested by Padfield & Lee^7^ to determine the role of visual invariant in regulating a wide set of helicopter’s maneuvers, and by Pinder et al.^48^ to test a visual interface developed to improve the regulation and decision-making of aircraft pilots during take-off and landing. The results obtained here on participants with no piloting experience prove that the present design succeeds in conveying information about the deck-landing possibilities. Experts can be expected to be at least as good as novices at understanding and using EID^49^. In addition, the exact nature of the information conveyed was established in a careful work domain analysis involving expert pilots. The present design will therefore probably convey information to experts as well. In short, both the informational content and the form of the Augmentation can be logically expected to ensure a good level of performance to more experienced pilots. The benefits of our Augmentation would perhaps be less obvious with experts because they would be more able to detect the affordances at work with the naked eye, to be more sensitive to high-order affordances ^31^, to make better decisions and regulate their actions more successfully ^50^ than novices. If experts’ performances per se would therefore not be greatly improved by our Augmentation (since they already perform well under non-augmented conditions), our interface would still no doubt have other more subtle benefits when used by expert pilots, such as reducing their workload^51^ and improving their error detection and situation awareness^49^, which would reduce the fatigue occasioned by this highly demanding task. These benefits will in turn improve these pilots’ ability to repeat safe landings despite having to cope with lengthy operations, stress, and even technical problems.

### Towards real-life applications

The first step in filling the gap between the present Augmentation designed to assist the decision-making process and future developments dealing with more ecological situations would require to continuously control the helicopter’s lift via the collective stick during the descent. The design of the visual Augmentation would therefore have to be reworked accordingly. This would first serve to test the relevance of the affordance model for deck-landing-ability to capture participants' ability to regulate their descent. Secondly, it would test the hypothesis that the coupling behavior previously observed^42^ may be exploratory behavior. We therefore expect that participants allowed to continuously control the collective stick would either naturally show this coupling behavior and/or produce better performances when forced to do so. These future results would be in line with the idea that perception of affordance is better under moving than stationary conditions^52^.

The second step in dealing with more ecological situations could secondly be achieved by improving the realism of the helicopter and ship movements so as to simulate the real-life dynamics more accurately. In the present study, the ship’s dynamics were simulated with a sinusoid, and the helicopter dynamics, using elementary lift-gravity models (cf. Equation 1). The current Augmentation would certainly benefit from incorporating finer helicopter and ship models. For example, the fidelity of helicopter models could be improved by including either linear or nonlinear dynamic features^53^. Methods for improving the ship dynamics on the other hand could be based on filter analogies or deterministic models based on ship and forthcoming encountered wave properties^54^.

The third step, including other constraints than the touchdown velocity constraint ${V}_{td}$ in the design of this Augmentation would greatly improve this approach for dealing with real-life situations. Balance and precision constraints, which are related to the roll, transversal and forward movements of the ship, are additional aspects worth taking into consideration when modeling the affordance and designing the Augmentation. Work domain analyses based on reviews of the literature^1,2,4,55^, technical reports^56^ and pilots’ interviews have suggested in fact that the deck-landing-ability is a multidimensional affordance^57^ which depends not only on the touchdown velocity constraints.

## Conclusions

The visual Augmentation we designed taking an ecological approach enhanced the safety of participants with no piloting experience by sharpening their perception of the *deck-landing-ability affordance*. Enabling to continuously control the helicopter’s lift in an immersive simulator in order to study the information pickup through exploratory movements will be the next step in our experimental program.

## Data Availability

The datasets generated by the laptop simulation and analyzed in the present study are available in the “HelicopterShipLanding-EID-decision_data” repository, https://github.com/AntoineHPMORICE/HelicopterShipLanding-EID-decision_data.
